# 323. Distribution of Pathogens in Coinfections of Patients Admitted with COVID-19

**DOI:** 10.1093/ofid/ofab466.525

**Published:** 2021-12-04

**Authors:** Srilatha Neshangi, Budder Siddiqui, Sarah Tran, Phillip Zhang

**Affiliations:** 1 Augusta University Medical Center, Augusta, Georgia; 2 Augusta University, Augusta, Georgia; 3 Medical College of Georgia, Augusta, Georgia

## Abstract

**Background:**

Patients who are admitted to the hospital with Coronavirus Disease 2019 (COVID-19) often have protracted hospitalizations complicated by bacterial or fungal co-infections. This also raises the question whether there is some feature of COVID-19 that predisposes to development of specific co-infections. To begin answering that question, we sought to review the distribution of microorganisms identified in bacterial and respiratory cultures in patients admitted with COVID-19.

**Methods:**

In a retrospective review of all patients admitted with COVID-19 in the year 2020 at a single academic tertiary medical facility, all positive blood and respiratory cultures were reviewed. Common contaminants were removed. Duplicate growth of the same organism within the same patient was not counted as a separate event.

**Results:**

787 patients were admitted with COVID-19 for the specified time frame. There were 131 and 147 unique events of documented bacterial or fungal growth seen in blood cultures and respiratory tract cultures, respectively. The most commonly identified organism in blood cultures was *Staphylococcus aureus* (3.94% of patients with COVID-19), followed closely by *Enterococcus* (2.41%), *Klebsiella* (1.65%), and *Escherichia* (1.27%). *Staphylococcus aureus* was also the most frequently isolated organism in respiratory cultures (7.24% of patients with COVID-19), followed by *Pseudomonas* (3.43%), *Klebsiella* (1.78%), *Serratia* (0.89%), and *Stenotrophomonas* (0.89%).

**Conclusion:**

This suggests that the distribution of pathogens implicated in coinfections in this patient population may not be substantially different from what might be expected in patients admitted for reasons outside of COVID-19. Further investigation with a larger patient population would provide more generalizable data, including patients admitted for reasons outside of COVID-19.

**Disclosures:**

**All Authors**: No reported disclosures

